# The effect of hypoxia on PD-L1 expression in bladder cancer

**DOI:** 10.1186/s12885-021-09009-7

**Published:** 2021-11-25

**Authors:** Vicky Smith, Debayan Mukherjee, Sapna Lunj, Ananya Choudhury, Peter Hoskin, Catharine West, Tim Illidge

**Affiliations:** 1 Division of Cancer Sciences, University of Manchester, M20 4BX Manchester, UK; 2 The Christie NHS Foundation Trust, Manchester, UK; 3 Manchester Academic Health Science Centre, Manchester, UK

**Keywords:** hypoxia, HIF, PD-L1, Tumour microenvironment, Immunosuppression, TIME, Cell density, Bladder cancer, MIBC

## Abstract

**Introduction:**

Recent data has demonstrated that hypoxia drives an immunosuppressive tumour microenvironment (TME) via various mechanisms including hypoxia inducible factor (HIF)-dependent upregulation of programmed death ligand 1 (PD-L1). Both hypoxia and an immunosuppressive TME are targetable independent negative prognostic factors for bladder cancer. Therefore we sought to investigate whether hypoxia is associated with upregulation of PD-L1 in the disease.

**Materials and methods:**

Three human muscle-invasive bladder cancer cell lines (T24, J82, UMUC3) were cultured in normoxia (20% oxygen) or hypoxia (1 and 0.1% oxygen) for 24 h. Differences in PD-L1 expression were measured using Western blotting, quantitative polymerase chain reaction (qPCR) and flow cytometry (≥3 independent experiments). Statistical tests performed were unpaired t tests and ANOVA. For in silico work an hypoxia signature was used to apply hypoxia scores to muscle-invasive bladder cancers from a clinical trial (BCON; *n* = 142) and TCGA (*n* = 404). Analyses were carried out using R and RStudio and statistical tests performed were linear models and one-way ANOVA.

**Results:**

When T24 cells were seeded at < 70% confluence, there was decreased PD-L1 protein (*p* = 0.009) and mRNA (*p* < 0.001) expression after culture in 0.1% oxygen. PD-L1 protein expression decreased in both 0.1% oxygen and 1% oxygen in a panel of muscle-invasive bladder cancer cells: T24 (*p* = 0.009 and 0.001), J82 (*p* = 0.008 and 0.013) and UMUC3 (*p* = 0.003 and 0.289). Increasing seeding density decreased PD-L1 protein (*p* < 0.001) and mRNA (*p* = 0.001) expression in T24 cells grown in both 20 and 1% oxygen. Only when cells were 100% confluent, were PD-L1 protein and mRNA levels higher in 1% versus 20% oxygen (*p* = 0.056 and *p* = 0.037). In silico analyses showed a positive correlation between hypoxia signature scores and PD-L1 expression in both BCON (*p* = 0.003) and TCGA (*p* < 0.001) cohorts, and between hypoxia and IFNγ signature scores (*p* < 0.001 for both).

**Conclusion:**

Tumour hypoxia correlates with increased PD-L1 expression in patient derived bladder cancer tumours. In vitro PD-L1 expression was affected by cell density and decreased PD-L1 expression was observed after culture in hypoxia in muscle-invasive bladder cancer cell lines. As cell density has such an important effect on PD-L1 expression, it should be considered when investigating PD-L1 expression in vitro.

**Supplementary Information:**

The online version contains supplementary material available at 10.1186/s12885-021-09009-7.

## Introduction

Bladder cancer is the tenth most common cause of cancer death in the UK with an overall 10-year survival rate of only 46% in England [1]. Stages two, three and four are classified as muscle-invasive bladder cancer (MIBC) for which the five-year survival rates are 45, 40 and 10% respectively [2].

The two definitive treatment approaches for MIBC are cystectomy, or radiotherapy with a radiosensitiser. Neoadjuvant chemotherapy can be given with either, but has a limited contribution to improved survival [3]. Immunotherapy for bladder cancer has an extensive history, with the first immunotherapy (Bacillus Calmette–Guérin) for non-MIBC being approved in 1990 [4]. In the last 5 years, six new immunotherapies were approved for advanced urothelial carcinoma, most targeting the PD-1/PD-L1 pathway. However, despite initial successes the rates of durable responses remain low with generally only around one in five patients showing a sustained response [5].

An immunosuppressive tumour microenvironment (TME) is known to be a negative prognostic factor contributing to recurrence of disease and tumour progression [6]. Hypoxia is a common feature of many solid tumours and is also an adverse prognostic factor in bladder cancer [7, 8]. Both an immunosuppressive TME and hypoxia contribute to the failure of radiotherapy [9, 10]. Therefore, the relationship between immunosuppression and hypoxia is of interest when developing biomarkers to guide the management of bladder cancer.

Hypoxia inducible factor (HIF1)-1α is a transcription factor that regulates a plethora of genes in response to decreased oxygen availability [11]. Recently, it was shown that hypoxia-induced gene changes affect tumour immune responses and contribute towards an immunosuppressive TME [12]. The mechanisms involved include direct effects on immune cells that alter their functions and indirect effects due to altered cytokine and chemokine expression that impact the recruitment and migration of immune cells [13, 14].

Specifically, hypoxia via HIF1α was shown to increase expression of the immune checkpoint gene programmed death - ligand 1 (PD-L1) in human and mouse cancer cell lines [15, 16]. The effect of hypoxia on PD-L1 expression in bladder cancer cells has not previously been reported.

Here we aimed to investigate the effects of hypoxia on PD-L1 expression in bladder cancer using in vitro and in silico approaches.

## Materials and methods

### Cell lines

Human MIBC cell lines (T24, J82, UMUC3) were cultured in Eagle’s Minimum Essential Media (Gibco; ThermoFisher Scientific, Loughborough, UK) supplemented with 10% foetal bovine serum (Gibco) and L-glutamine (2 mM; Gibco). Cell lines were authenticated by the Cancer Research UK Manchester Institute core facilities services every 6 months and tested for mycoplasma monthly.

### Exposure to hypoxia

Cells were seeded at a density of 1 × 10^5^ per well, unless otherwise stated, onto 6-well cell culture plates (Corning™ Costar™, Leicestershire, UK) in a humidified atmosphere of air plus 5% CO_2_ (here termed normoxia) and left to adhere for 24 h. Some plates were then transferred to a hypoxia cabinet (Baker-Russkin®, Bridgend, Wales), washed with phosphate buffered saline (PBS; made by in-house services) and fresh media added. Both the PBS and fresh media were pre-equilibrated to the required oxygen concentration for 24 h. The other plates were processed in the same way but in normoxia.

### Treating cells with pharmacological agents

Human recombinant interferon gamma (IFNγ) (Peprotech, London, UK) was reconstituted in dimethyl sulphoxide (DMSO) to make a working stock solution of 20 μg/ml. It was added directly to media culture at the same point of processing as described above to a working concentration of 10 ng/ml.

### Western blotting

Cells were lysed in situ by adding lysis buffer solution (Cell Signalling Technology, London, UK) and protease inhibitor cocktail (Cell Signalling Technology) and placing on ice for 10 min. Samples were then sonicated at 10 amplitude microns for 10 s using a Soniprep 150 (Measuring and Scientific Equipment, London, UK) before centrifugation at 14,000 G for 15 min. Protein concentrations of the lysates were measured using a BCA Protein Assay kit (ThermoFisher Scientific, Loughborough, UK), and the solution resolved on 4–20% Tris-Glycine Protein Gels (ThermoFisher Scientific) prior to transferring to a nitrocellulose membrane (Bio-Rad, Watford, UK). The membrane was then incubated with the primary and secondary antibodies with PBS washes in between. Table 1 lists antibody suppliers and concentrations.

**Table 1:** Concentrations and supplier information of antibodies used for Western blotting

Target	Raised in	Dilution	Supplier	Code
PD-L1	Rabbit	1:2000	Cell Signalling Technology	13,684
HIF1α	Mouse	1:500	BD	610,959
GAPDH	Rabbit	1:2500	Cell Signalling Technology	2118
Mouse-HRP	Goat	1:7500	Invitrogen	62–6520
Rabbit-HRP	Goat	1:5000	Invitrogen	65–6120

### Real-time PCR

RNA was extracted using a Qiagen RNeasy Mini Kit according to the manufacturer’s protocol and quantified using a NanoDrop™ (ThermoFisher Scientific). cDNA was obtained using OmniScript RT Kit (Qiagen, Manchester, UK) with random hexamer primers (ThermoFisher Scientific) and RNase Inhibitor (New England Biolabs, Hitchin, UK). Primers were designed by Primer-BLAST and Primer3 software, checked using Beacon Design Software (Premier Biosoft) and made by Eurofins Genomics (Ebersberg, Germany) before being resuspended in Tris – EDTA (TE) buffer. Table 2 lists primer sequences. Serial dilutions of primers were used for validation experiments and primer mixes were made with SYBR Green Master Mix (ThermoFisher Scientific). 384-well PCR plates were run using a QuantStudio 5 Real-Time PCR System and appropriate controls.

**Table 2:** Primer sequences and their properties used for qPCR

Primer	Sequence	Tm (°C)	GC-content (%)
PD-L1 forward	TATGGTGGTGCCGACTACAA	57.3	50
PD-L1 reverse	TGGCTCCCAGAATTACCAAG	57.3	50
SDHA forward	CATCCACTACATGACGGAGCA	59.8	52.4
SDHA reverse	ATCTTGCCATCTTCAGTTCTGCTA	59.3	41.7

### Flow cytometry

Cells were trypsinised, placed in fluorescence-activated cell sorting (FACS) tubes (Corning, Flintshire, UK), washed in FACS buffer (1% FBS in PBS), centrifuged and resuspended in a series of solutions with FACS buffer washing steps in between. In order, the cells were resuspended in a live/dead fixable cell stain (1:1000; Invitrogen; ThermoFisher Scientific), human FcR blocking solution (1:100; Miltenyi Biotec, Woking, UK), then either PD-L1 PE antibody (1:100; Invitrogen) or control PE mouse IgG1 k isotype antibody (1:100) (BD Pharmigen, CA, USA) before being fixed in 1% paraformaldehyde. Samples were acquired on a LSRII flow cytometer (BD Biosciences, CA, USA) with 10,000 viable cells collected per sample. Flow cytometry was performed in either duplicate or triplicate wells as stated and each well was split into two FACS tubes for immunoglobulin control alongside target of interest. The data collected were then processed using FlowJo software (version 10.6.1). Table 3 lists reagent details.

**Table 3:** Concentrations and supplier details of reagents used for flow cytometry

Name	Details	Dilution	Supplier	Code
Live/dead stain	Diluted in PBS	1:1000	Invitrogen	L34973
Fc blocking	Diluted in FACs buffer	1:100	Miltenyi Biotec	130–059-901
PD-L1 antibody	PE conjugated	1:100	Fisher Scientific	12–5983-41
IgG antibody	PE conjugated	1:100	BD Pharmigen	554,121

### Bioinformatics

BCON is a clinical trial registered as CRUK/01/003 of which the details and conclusions are outlined in the initial findings report [17]. Gene expression data from the BCON cohort were obtained as previously described [18] and the TCGA-BLCA data were obtained using the R packages “TCGAUtils” and “curatedTCGAData”. Both datasets were filtered to include only tumours of a known stage and stage 2 and above. Analyses were carried out using R (version 4.0.3) and RStudio (version 1.3).

### Statistical analysis

In vitro data were tested by unpaired t test or ANOVA using GraphPad Prism 8 software. In silico data were tested by linear regression, Pearson’s correlation and ANOVA using R and RStudio.

## Results

### Hypoxia decreases expression of PD-L1 in T24 human bladder cancer cells

**Fig. 1:**
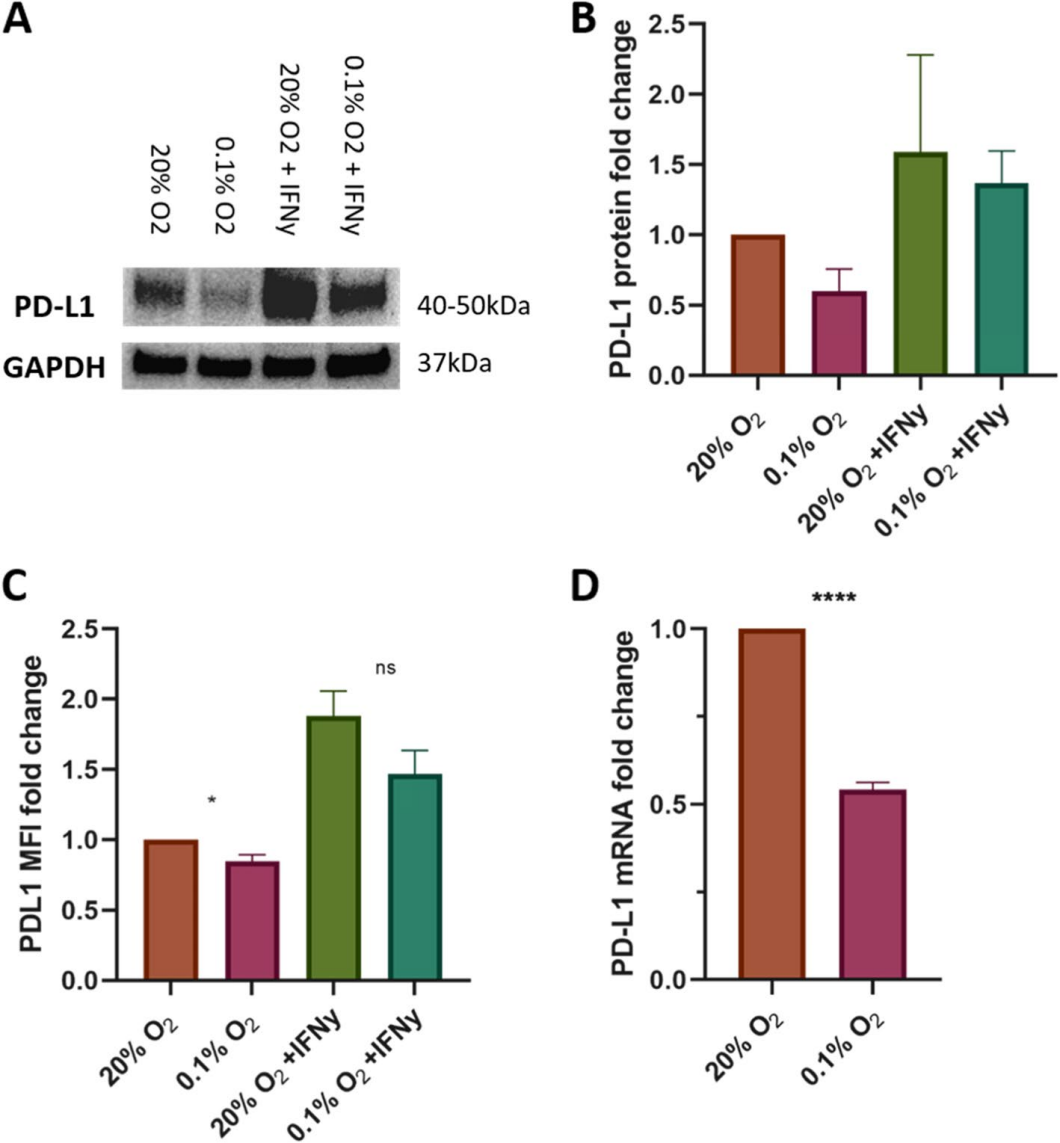
Hypoxia (0.1%) decreases the expression of PD-L1 in T24 bladder cancer cells. PD-L1 expression decreased in T24 cells after 24 h culture in 0.1% oxygen. IFNγ stimulation increased expression of PD-L1 in 20% O_2_, but the IFNγ-driven increase was attenuated in cells grown in 0.1% O_2_. Cells were seeded and left to adhere for 24 h before placing into a hypoxia chamber for 24 h and/or 10 ng/ml IFNγ added to the culture media. **A)** Western blotting shows the change in protein levels of PD-L1 with GAPDH used as a loading control. Three independent experiments were carried out and a representative image is shown. **B)** Quantification by densitometry analysis was performed using ImageJ by calculating the relative densities of both the loading control and the samples to the control untreated lane. These values were then scaled to the relative density values to find adjusted relative values from three independent experiments. **C)** Flow cytometry shows the change in surface expression of PD-L1. Data are the mean ± standard error of the mean (SEM) of the mean fluorescence intensity of 10,000 viable cells from replicates of four independent experiments normalised to normoxia untreated condition to show the relative fold change. **D)** qPCR shows changes in mRNA levels relative to the mRNA levels of T24 cells cultured in 20% O_2_. Data are the mean ± standard error of the mean (SEM) from three independent experiments plated in triplicate with differences calculated using the delta-delta Ct method relative to the expression of reference gene SDHA. Statistical tests are unpaired t tests performed in GraphPad Prism with *p* values represented as follows: ns = not significant, * < 0.05, ** < 0.01, *** < 0.001, **** < 0.0001

To investigate the effects of hypoxia on PD-L1 expression in T24 MIBC cells they were cultured at 0.1% oxygen and 20% oxygen and the differences in PD-L1 expression examined. PD-L1 expression significantly decreased in hypoxia (0.1% oxygen for 24 h) at both the RNA (qPCR) and protein (Western blotting, flow cytometry) level in T24 human bladder cancer cells (Fig. 1). IFNγ is a known stimulant of PD-L1 and its addition led to the expected stimulation of PD-L1 [19]. However, when IFNγ stimulated cells were cultured in hypoxia, the IFNγ-driven PD-L1 increase was reduced (Fig. 1). This finding further highlights the negating effects of hypoxia on PD-L1 expression in T24 cells. HIF1α protein expression was present in all the samples cultured in hypoxia (0.1% oxygen) and absent in those under normoxia, confirming the cells are responding to the hypoxic conditions (Supplementary Fig. 1). Comparison of the proportion of viable cells between samples showed hypoxia did not induce excessive cell death (Supplementary Fig. 2).

### Both 0.1 and 1% hypoxia decreases PD-L1 expression in a panel of human muscle-invasive bladder cancer cells

**Fig. 2:**
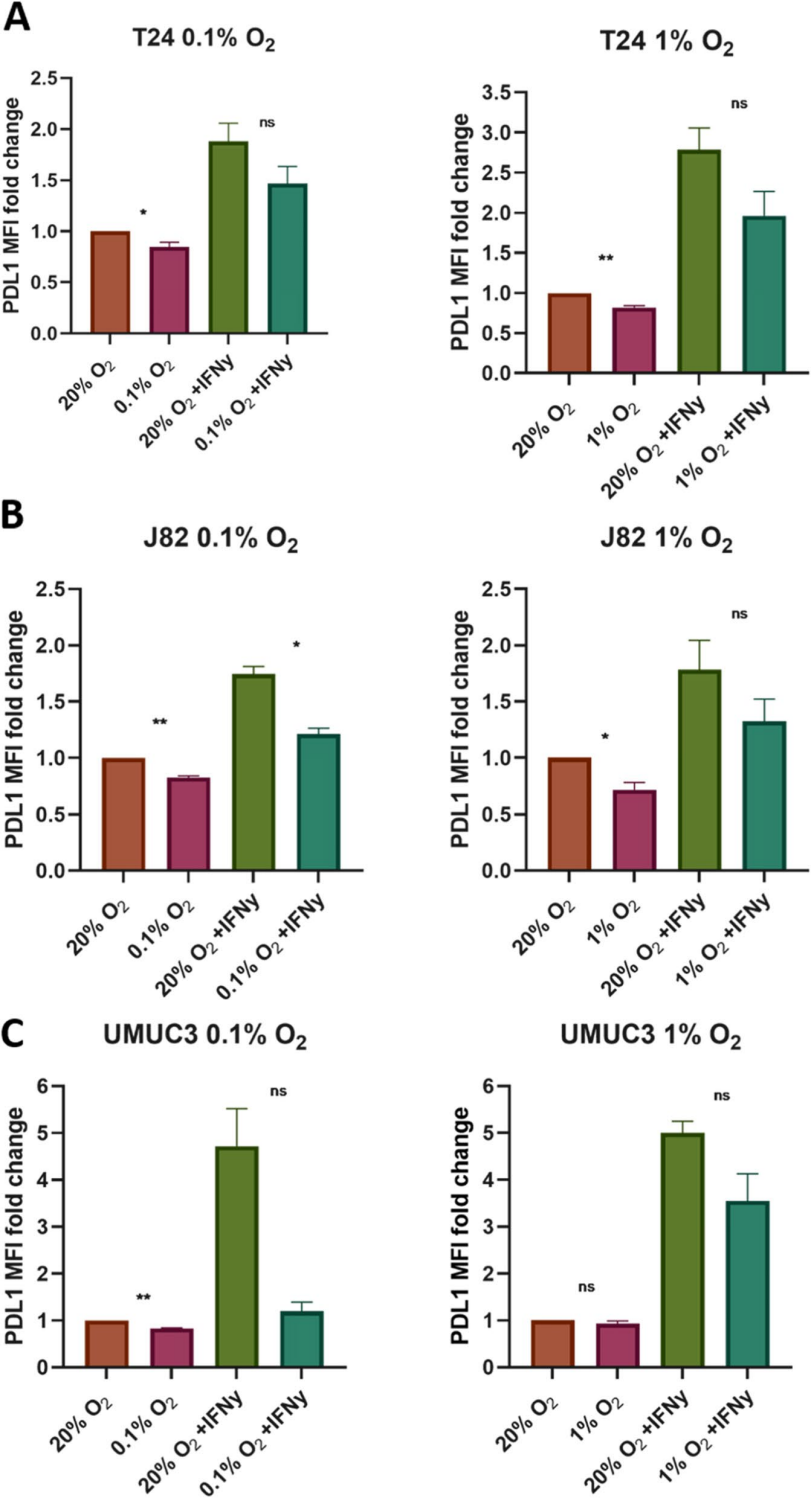
Hypoxia (0.1 and 1%) decreases PD-L1 expression in a panel of human bladder cancer cells. Flow cytometry analyses show the surface expression of PD-L1 decreases after culture in hypoxia across a panel of human bladder cancer cells. Culture in 0.1 and 1% O_2_ for 24 h decreases the expression of PD-L1 compared with the levels present when cultured under 20% O_2_ in T24 (**A)**, J82 (**B**), UMUC3 (**C**) bladder cancer cells. Cells were seeded and left to adhere for 24 h before placing in 0.1% or 1% O_2_ for 24 h and 10 ng/mL IFNγ added to the relevant wells. Data are the mean ± standard error of the mean (SEM) of the mean fluorescence intensity of 10,000 viable cells from replicates of at least two independent experiments normalised to the normoxia untreated condition to show the relative fold change. Statistical tests are unpaired t tests performed in GraphPad Prism with *p* values represented as follows: ns = not significant, * < 0.05, ** < 0.01, *** < 0.001, **** < 0.0001

To investigate whether the decrease in PD-L1 might be cell line or oxygen concentration dependent, we investigated two other human MIBC cell lines (J82 and UMUC3) and a less severe level of hypoxia (1% oxygen). There was a consistent significant decrease in PD-L1 protein expression after exposure to hypoxia in all three bladder cancer cell lines and at both 0.1 and 1% oxygen concentrations (Fig. 2). Across the three cell lines there was also a continued attenuation of the IFNγ-driven PD-L1 induction in hypoxia.

### PD-L1 levels decrease as cell density increases and a PD-L1 increase in hypoxia occurs only when cells are highly confluent

**Fig. 3:**
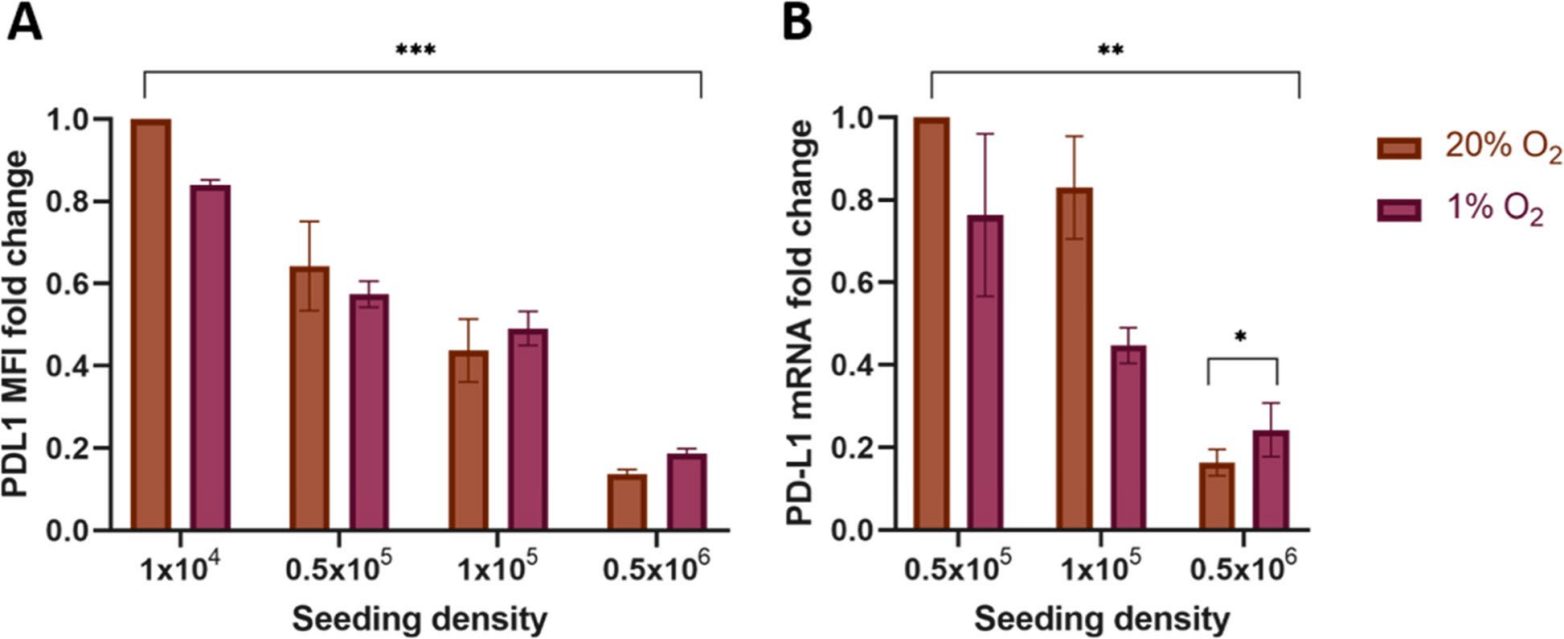
PD-L1 expression decreases with increasing cell density and hypoxia-induced PD-L1 increase occurs only in high-density cells. As T24 cell seeding density increases, the protein and mRNA expression of PD-L1 decrease in both normoxia and hypoxia conditions (*p* < 0.001 and *p* = 0.0011 respectively). When the cells are cultured at higher densities (100% confluence) PD-L1 protein and mRNA expression increases after culture in hypoxia compared with normoxia (*p* = 0.05576 and *p* = 0.03721 respectively). Cells were counted and seeded into six-well plates at different densities, left to adhere for 24 h and then incubated for 24 h. **A)** Flow cytometry shows the change in surface expression of PD-L1. Data are the mean ± standard error of the mean (SEM) of the mean fluorescence intensity of 10,000 viable cells from replicates of three independent experiments normalised to normoxia untreated condition to show the relative fold change. **B)** qPCR shows changes in mRNA levels relative to the mRNA levels of T24 cells cultured in a 20% O_2_ incubator. Data are the mean ± standard error of the mean (SEM) from three independent experiments plated in triplicate and the difference calculated using the delta-delta Ct method relative to the expression of reference gene SDHA. Statistical tests are linear models and ANOVA performed using R and RStudio with *p* values represented as follows: ns = not significant, * < 0.05, ** < 0.01, *** < 0.001, **** < 0.0001

The effects of cell density on hypoxia-induced changes in PD-L1 expression were explored in T24 cells. This cell line was used due to its fast proliferation rate, with a doubling time of around 20 h [20, 21], which facilitated the assessment of increasing cell density. As we showed similar effects in both 0.1 and 1% hypoxia we took forward the less severe 1% hypoxia for further experiments to minimise stress on the cells. Density gradient experiments showed PD-L1 protein and mRNA expression decreased significantly with increasing cell density in both normoxia and hypoxia (Fig. 3). A significant hypoxia-induced increase in PD-L1 expression was only seen when the cells were seeded at the highest densities (Fig. 3). Increasing cell density had no effect on viability (Supplementary Fig. 3).

### PD-L1 gene expression correlates positively with hypoxia in muscle-invasive bladder cancer patients

**Fig. 4:**
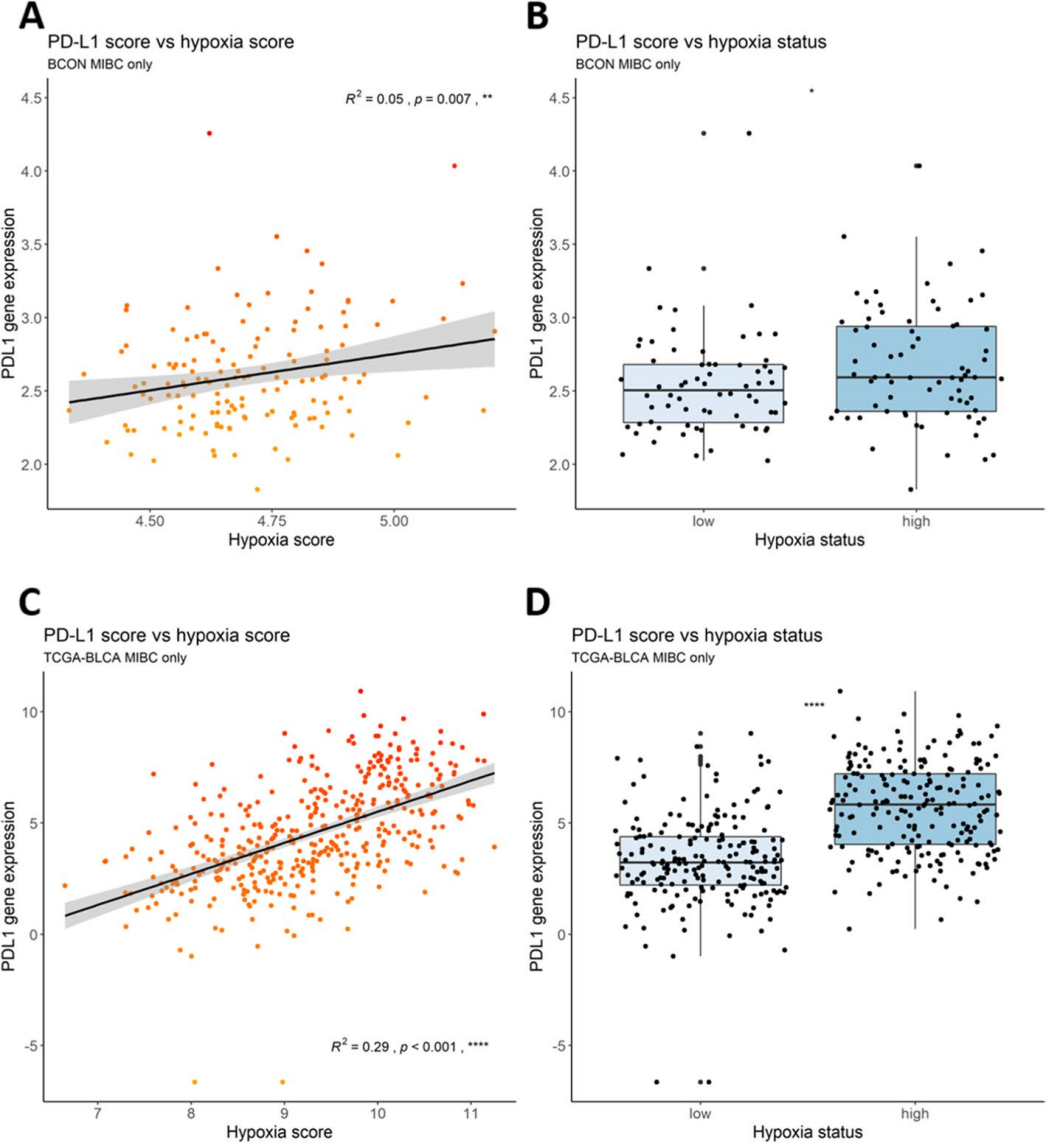
In silico analyses show a positive association between hypoxia and PD-L1 in muscle-invasive bladder tumours. Analyses involved microarray data from the BCON trial normalised previously using aroma package and filtered to include only tumours stage 2 and above (*n* = 142) and the TCGA-BLCA RNASeq2GeneNorm dataset downloaded using the Bioconductor package TCGAutils and filtered to include only tumours of a known stage 2 and above (*n* = 404). Hypoxia scores were applied to each tumour sample using a previously published 24-gene bladder cancer hypoxia signature. Hypoxia signature scores were plotted against the expression of PD-L1 in **A**) BCON and **C**) TCGA cohorts. R^2^ values were calculated using Pearson’s correlation coefficient and the *p* values represent a linear model analysis. Tumours were stratified into hypoxia low or high using the median of the hypoxia scores for each cohort, and plotted against the PD-L1 expression in the **B)** BCON and **D)** TCGA cohorts. *P* values were calculated using one way ANOVA between the two groups. p values are represented as follows: ns = not significant, * < 0.05, ** < 0.01, *** < 0.001, **** < 0.0001

To elucidate further the potential relationship and clinical relevance between hypoxia and PD-L1, in silico analyses were performed using gene expression datasets from two bladder cancer cohorts. A bladder cancer specific hypoxia signature previously published was used to assign hypoxia scores, which were then correlated with the expression of PD-L1 (CD274) [18]. A significant positive correlation was seen between hypoxia signature scores and the expression of PD-L1 in both cohorts (Fig. 4A, B). The median hypoxic score across each cohort stratified patients into low and high hypoxia groups. In both cohorts, the high hypoxia tumours had a significantly higher expression of PD-L1 compared with low hypoxia tumours (Fig. 4C, D).

### Hypoxia and IFNγ-signalling signature scores correlate positively in muscle-invasive bladder cancer patients

**Fig. 5:**
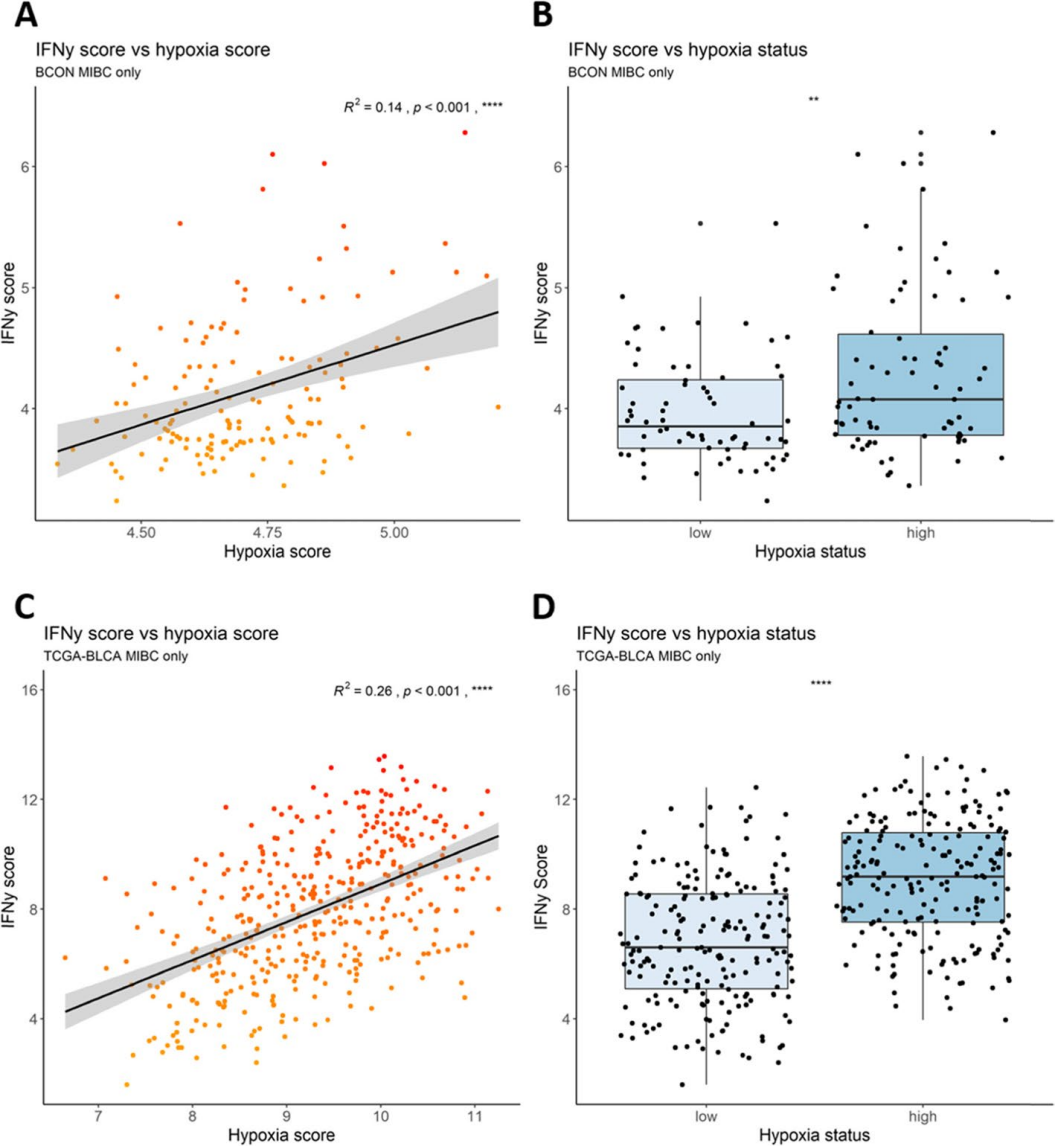
In silico analyses show a positive association between hypoxia and IFNγ-signalling in muscle-invasive bladder tumours. Hypoxia scores were applied to tumours from BCON and TCGA cohorts filtered to include stage 2 and above only. A published 6-gene IFNγ gene signature was used to attribute an IFNγ score to each tumour. These scores were plotted against the hypoxia scores in the **A)** BCON and **C)** TCGA cohorts. R^2^ values were calculated using Pearson’s correlation coefficient and the *p* values represent a linear model analysis. Tumours were stratified into hypoxia low or high using the median of the hypoxia score from each cohort, and plotted against the IFNγ signature score in the **B)** BCON and **D)** TCGA cohorts. *P* values were calculated using one way ANOVA between the two groups. p values are represented as follows: ns = not significant, * < 0.05, ** < 0.01, *** < 0.001, **** < 0.0001

Finally, a published IFNγ gene signature representing extent of IFNγ signalling was used to attribute IFNγ scores to the BCON and TCGA cohorts [22]. There were positive correlations between hypoxia and IFNγ signature scores (Fig. 5A, B). The tumours were again stratified into low and high hypoxia and in both cohorts the high hypoxia tumours showed significantly higher IFNγ scores compared with the low hypoxia tumours (Fig. 5C, D).

## Discussion

Several findings emerged from this study. First, hypoxia decreased PD-L1 expression and abrogated IFNγ-induced increases in PD-L1 in bladder cancer cells. Second, PD-L1 expression decreased with increasing cell seeding density, which was pronounced in cells cultured in normoxia versus hypoxia. Third, a hypoxia-induced increase in PD-L1 was only seen with the highest cell seeding densities when there was a marked down-regulation of PD-L1 in cells grown in normoxia. Fourth, PD-L1 gene expression, as well as IFNγ-signalling expression, correlated positively with hypoxia in bladder cancers in silico.

The observation that hypoxia decreases PD-L1 expression initially appears to conflict with conclusions made in published literature. Noman et al. [16] concluded that hypoxia induced an upregulation of PD-L1 in a panel of murine and human cell lines. However, the finding appears to be tissue-type specific as only one (B16-F10) of four murine cell lines (B16-F10 melanoma; LLC lung; CT26 colon; 4 T1 mammary) studied showed an increase in the percentage of PD-L1 positive cells after culture in 0.1% oxygen. There was a minimal increase at 72 h in the lung, no change in the colon, and a non-significant decrease in the mammary cell line. In the human cell lines, there was a marked hypoxia-induced increase in PD-L1 in melanoma (T1, M4T), a small significant increase in lung (IGR-Heu), and no change in breast (MCF-7). In addition, all the hypoxia results (24 h, 48 h, 72 h) were compared with 72 h normoxia data. Interestingly, across the cell lines, there were no significant changes observed at 24 h, except for the murine melanoma cell line, and most of the significant changes were seen at the 72 h time-point. As cell density will increase at the later time-point, the results might be affected by the cell density effect we found here.

Barsoum et al. [15] also reported hypoxia-induced upregulation of PD-L1 expression. They incubated cells in 0.5% oxygen for 24 h and showed an increase in PD-L1 expression in human prostate (DU145) and breast (MDA-MB-231) cancer cells. The authors stated that all their experiments were conducted using cultures that did not exceed 70% confluence. The study also used siRNA knockdown experiments to show that PD-L1 upregulation was HIF1α dependent. HIFs bind to gene promoter regions known as hypoxia response elements (HREs). In 2011, Schödel et al. used chromatin immunoprecipitation sequencing (ChIPseq) to perform high resolution mapping of HIF binding sites in MCF-7 cells; PD-L1 was not in their 300+ list of high stringency HIF1 and HIF2 binding sites [23]. Given that Noman et al. also saw no effect of hypoxia on the expression of PD-L1 in MCF-7 cells, but Barsoum et al. identified HREs in the PD-L1 gene in DU145 cell lines as well as an hypoxia-induced increase in PD-L1 expression, it is possible that the hypoxia-induced upregulation of PD-L1 is tissue specific [15, 16]. To our knowledge this is the first documentation of the effects of hypoxia on PD-L1 expression in bladder cancer cell lines. The current literature outlines a discrepancy between the effects of hypoxia on the expression of PD-L1 across cell lines of different tissue origins and further comprehensive characterisation across an extensive panel of other cancer cell lines is required for more definitive conclusions.

Hypoxic cells preserve energy by reducing metabolic processes via HIF regulation of various genes [11]. Decreases in cellular metabolism also occur as a result of increasing cell density, and a recently published report underlines the importance of considering cell density in in vitro experiments and how cell density affects cellular metabolic changes [24, 25]. Therefore, the PD-L1 decrease we found in cells cultured in hypoxia could be due to reduced cellular metabolism. Furthermore, cells proliferate faster in normoxia than in hypoxia due to having higher cellular metabolism. Therefore, in the highly dense cells, a cell density-mediated reduction in metabolism should occur faster and be more pronounced in normoxia. The observed effect, therefore, might be an abrogated decreased PD-L1 expression occurring in the densely packed hypoxic conditions, rather than a true cellular hypoxia-driven increase and warrants further investigation. In support of this suggestion, several publications showed that increasing cell density results in decreased expression of cell surface markers including transforming growth factor beta (TGF-b) receptor in fibroblast cells, epidermal growth factor receptors (EGFRs) in breast cancer cells, and tumour necrosis factor (TNF) receptors in HeLa epithelial and myeloid HL-60 cell lines [26–28].

Increasing cell density leads to contact inhibition and cell cycle arrest via the Hippo/YAP pathway [29], and interacts with multiple intracellular signalling pathways [30–32] that regulate cyclin D expression [33]. PD-L1 expression is also affected directly by the cell cycle via cyclin D regulation [34] and via interactions with multiple cell signalling pathways, e.g., via PI3K/AKT, JAK/STAT3, WNT, NFkB and MAPK [35, 36]. This complex interplay between cell density, cell cycle, cell signalling and PD-L1 expression is yet to be fully elucidated and more research is needed to understand how hypoxia affects the interactions. Although we showed cell density affects the expression of PD-L1, we have not identified whether it is a direct effect, or due to cell density-mediated changes in cell cycle or cell signalling. The discrepancy in some of the in vitro results could potentially be explained by further investigations into the cell cycle and cell signalling pathways to determine how these affect the changes in PD-L1 expression in response to hypoxia.

To further explore the relationship between hypoxia and PD-L1 beyond the in vitro cell culture experiments we performed in silico analyses in patient tumours. The BCON trial randomised patients to receive either radiotherapy or radiotherapy plus hypoxia-modifying carbogen and nicotinamide (CON). TCGA-BLCA is a cohort of bladder cancer patients who underwent surgery and the gene expression dataset from these tumours is publicly available. The positive correlations seen between hypoxia signature scores and PD-L1 expression in both cohorts indicates that, despite our cell line findings, there is a relationship between the extent of hypoxia and increased PD-L1 expression in bladder cancer. This has implications for the treatment of MIBC whereby some patients may potentially benefit from a combination of hypoxia-modifying therapy with immunotherapy agents. In the same cohorts, we saw a positive correlation between hypoxia and IFNγ-signalling signature scores. IFNγ is known to stimulate PD-L1 and increased PD-L1 expression in the more hypoxic tumours could be a direct result of increased IFNy signalling [19]. Current in silico investigations have shown more immune infiltrates present as tumour hypoxia increases. As IFNγ is a central immune signalling molecule that is produced by many immune infiltrates, this provides an explanation for the increased IFNγ-signalling in the more hypoxic tumours. This suggestion needs investigating further. Taken together, our in vitro and in silico findings show that, although hypoxia-mediated cellular PD-L1 upregulation is not seen in bladder cancer cell lines, there is an overall increased expression of PD-L1 as tumour hypoxia increases in bladder cancer, which could be a result of increased IFNγ-signalling in the more hypoxic TMEs leading to an increased PD-L1 expression. These results highlight the importance of including patient sample analysis alongside cell line work when investigating immune-related contexts to provide translatable findings that account for the immune tumour microenvironment.

## Conclusions

In conclusion, we report for the first time that in bladder cancer cells the in vitro cell density affects PD-L1 expression in contrast to an absence of hypoxia-induced increase in PD-L1 expression. These findings underlie both the importance of cell density on the expression of PD-L1 in vitro and the need to address and document this in further publications. Our clinical data provide evidence that hypoxia may induce an immunosuppressive TME in bladder cancer and highlight the importance of further studies to investigate the underlying mechanisms.

## Supplementary Information

**Additional file 1: Supplementary Figure 1.** HIF1a is present in T24 cells cultured in hypoxia and absent when cultured in normoxia. Western blot showing the presence/absence of HIF1α across different experimental conditions alongside the changes in PD-L1 expression. GAPDH was used as an experimental loading control. Independent experiments were performed three times and a representative blot shown.

**Additional file 2: Supplementary Figure 2.** Hypoxia does not induce excessive cell death in T24 cells. Flow cytometry shows there is no excessive cell death induced by culture in 0.1% O_2_. A live/dead stain was incorporated into the assay, which only enters cells with compromised membranes. Gating around cells with no dye uptake and comparing with total population allows for the analysis of the proportion of viable cells. Data are the mean ± standard error of the mean (SEM) from at least three independent experiments performed in duplicates, of which each sample had 10,000 viable cells analysed.

**Additional file 3: Supplementary Figure 3.** Increased cell seeding density does not induce excessive cell death in T24 cells. Flow cytometry shows no increase in cell death neither by culture in 0.1% O_2_ nor as the cell seeding density increases. A live/dead stain was incorporated into the assay, which only enters cells with compromised membranes. Gating around cells with no dye present and comparing with total population allows for the analysis of the proportion of viable cells. Data are the mean ± standard error of the mean (SEM) from at least three independent experiments performed in duplicates, with 10,000 viable cells analysed per sample.

**Additional file 4: Supplementary Figure 4**. Tabulated data from the graphs shown in Fig. 2. This table shows the averages of normalised flow cytometry results comparing changes in PD-L1 expression across various experimental conditions.

**Additional file 5: Supplementary Figure 5.** Full length original and unprocessed Western blots shown in Fig. 1A. Western blot showing the presence/absence of A) PD-L1 and B) GAPDH across different experimental conditions. GAPDH was used as an experimental loading control. Independent experiments were performed at least three times and a representative blot is shown.

**Additional file 6: Supplementary Figure 6.** Full length original and unprocessed Western blots shown in Supplementary Fig. 1. Western blot showing the presence/absence of A) HIF1a, B) PD-L1 and C) GAPDH across different experimental conditions. GAPDH was used as an experimental loading control. Independent experiments were performed at least three times and a representative blot is shown.

## Data Availability

All raw data from in vitro experiments as well as further data supporting the chosen representative image presented in this published article and its supplementary files can be made available from the corresponding author on request. The TCGA-BLCA gene expression data are publically available and were obtained using the R package “curatedTCGAData” to download the RNASeq2GeneNorm assay from the disease code BLCA (BLCA_RNASeq2GeneNorm-20160128). The BCON clinical data are not publically available and belong to Peter Hoskin of the University of Manchester, who is to be contacted for the data to be made available on reasonable request.
