# Supplementary material for: The effect of hypoxia on PD-L1 expression in bladder cancer
Source: BMC Cancer. 2021 Nov 25;21:1271. doi: 10.1186/s12885-021-09009-7 (PMC8613983; doi:10.1186/s12885-021-09009-7)
Supplement: Supplementary file 1 — Additional file 1: Supplementary Figure 1. HIF1a is present in T24 cells cultured in hypoxia and absent when cultured in normoxia. Western blot showing the presence/absence of HIF1α across different experimental conditions alongside the changes in PD-L1 expression. GAPDH was used as an experimental loading control. Independent experiments were performed three times and a representative blot shown. [file 12885_2021_9009_MOESM1_ESM.docx]

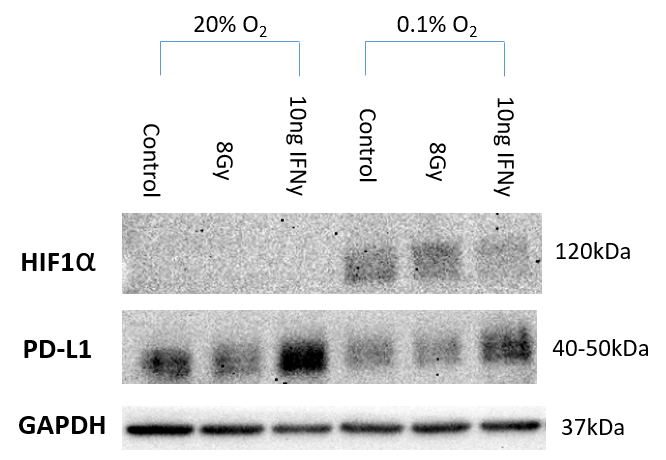


**Supplementary Figure 1. HIF1**α **is present in T24 cells cultured in hypoxia and absent when cultured in normoxia.** Western blot showing the presence/absence of HIF1α across different experimental conditions alongside the changes in PD-L1 expression across various experimental conditions. GAPDH was used as an experimental loading control. Independent experiments were performed three times and a representative blot shown.
