# Supplementary material for: The effect of hypoxia on PD-L1 expression in bladder cancer
Source: BMC Cancer. 2021 Nov 25;21:1271. doi: 10.1186/s12885-021-09009-7 (PMC8613983; doi:10.1186/s12885-021-09009-7)
Supplement: Supplementary file 2 — Additional file 2: Supplementary Figure 2. Hypoxia does not induce excessive cell death in T24 cells. Flow cytometry shows there is no excessive cell death induced by culture in 0.1% O2. A live/dead stain was incorporated into the assay, which only enters cells with compromised membranes. Gating around cells with no dye uptake and comparing with total population allows for the analysis of the proportion of viable cells. Data are the mean ± standard error of the mean (SEM) from at least three independent experiments performed in duplicates, of which each sample had 10,000 viable cells analysed. [file 12885_2021_9009_MOESM2_ESM.docx]

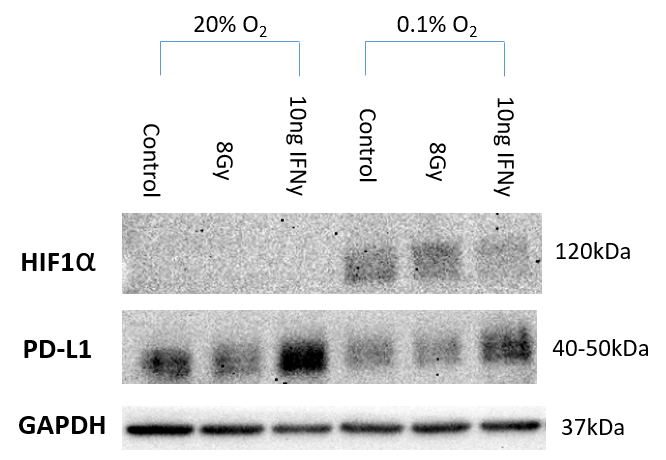


**Supplementary Figure 2. HIF1**α **is present in T24 cells cultured in hypoxia and absent when cultured in normoxia.** Western blot showing the presence/absence of HIF1α across different experimental conditions alongside the changes in PD-L1 expression. GAPDH was used as an experimental loading control. Independent experiments were performed three times and a representative blot shown.
