# Supplementary material for: The effect of hypoxia on PD-L1 expression in bladder cancer
Source: BMC Cancer. 2021 Nov 25;21:1271. doi: 10.1186/s12885-021-09009-7 (PMC8613983; doi:10.1186/s12885-021-09009-7)
Supplement: Supplementary file 3 — Additional file 3: Supplementary Figure 3. Increased cell seeding density does not induce excessive cell death in T24 cells. Flow cytometry shows no increase in cell death neither by culture in 0.1% O2 nor as the cell seeding density increases. A live/dead stain was incorporated into the assay, which only enters cells with compromised membranes. Gating around cells with no dye present and comparing with total population allows for the analysis of the proportion of viable cells. Data are the mean ± standard error of the mean (SEM) from at least three independent experiments performed in duplicates, with 10,000 viable cells analysed per sample. [file 12885_2021_9009_MOESM3_ESM.docx]

**
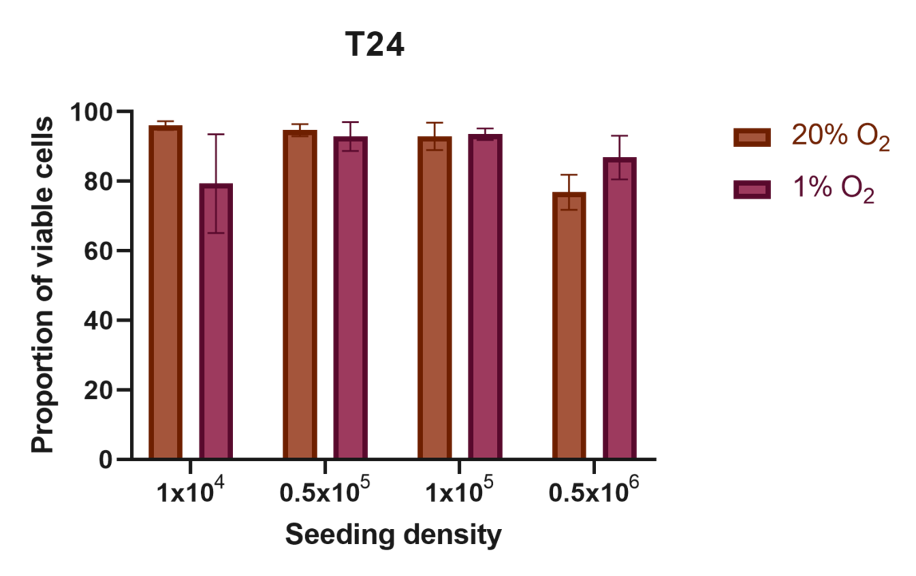
**

**Supplementary Figure 3. Increased cell seeding density does not induce excessive cell death in T24 cells.** Flow cytometry shows no increase in cell death neither by culture in 0.1% O_2_ nor as the cell seeding density increases. A live/dead stain was incorporated into the assay, which only enters cells with compromised membranes. Gating around cells with no dye present and comparing with total population allows for the analysis of the proportion of viable cells. Data are the mean ± standard error of the mean (SEM) from at least three independent experiments performed in duplicates, with 10,000 viable cells analysed per sample.
