# Supplementary material for: The effect of hypoxia on PD-L1 expression in bladder cancer
Source: BMC Cancer. 2021 Nov 25;21:1271. doi: 10.1186/s12885-021-09009-7 (PMC8613983; doi:10.1186/s12885-021-09009-7)
Supplement: Supplementary file 4 — Additional file 4: Supplementary Figure 4. Tabulated data from the graphs shown in Fig. 2. This table shows the averages of normalised flow cytometry results comparing changes in PD-L1 expression across various experimental conditions. [file 12885_2021_9009_MOESM4_ESM.docx]

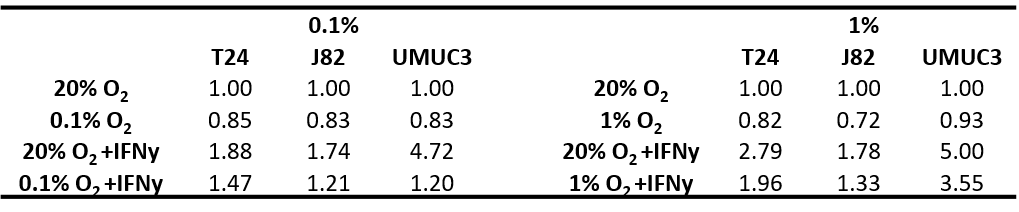


**Supplementary Figure 4**. **Tabulated data from the graphs shown in Figure 2**. This table shows the averages of normalised flow cytometry results comparing changes in PD-L1 expression across various experimental conditions.
