# Supplementary material for: The effect of hypoxia on PD-L1 expression in bladder cancer
Source: BMC Cancer. 2021 Nov 25;21:1271. doi: 10.1186/s12885-021-09009-7 (PMC8613983; doi:10.1186/s12885-021-09009-7)
Supplement: Supplementary file 5 — Additional file 5: Supplementary Figure 5. Full length original and unprocessed Western blots shown in Fig. 1A. Western blot showing the presence/absence of A) PD-L1 and B) GAPDH across different experimental conditions. GAPDH was used as an experimental loading control. Independent experiments were performed at least three times and a representative blot is shown. [file 12885_2021_9009_MOESM5_ESM.docx]

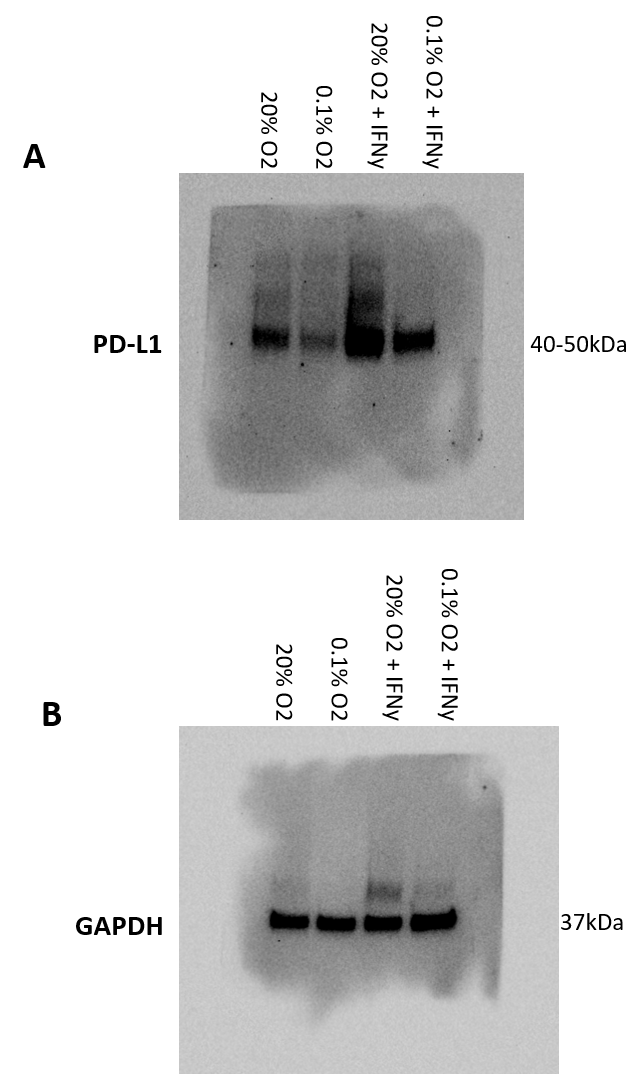


**Supplementary Figure 5. Full length original and unprocessed Western blots shown in Figure 1A.**  Western blot showing the presence/absence of **A)** PD-L1 and **B)** GAPDH across different experimental conditions. GAPDH was used as an experimental loading control. Independent experiments were performed at least three times and a representative blot is shown.
