# Supplementary material for: The effect of hypoxia on PD-L1 expression in bladder cancer
Source: BMC Cancer. 2021 Nov 25;21:1271. doi: 10.1186/s12885-021-09009-7 (PMC8613983; doi:10.1186/s12885-021-09009-7)
Supplement: Supplementary file 6 — Additional file 6: Supplementary Figure 6. Full length original and unprocessed Western blots shown in Supplementary Fig. 1. Western blot showing the presence/absence of A) HIF1a, B) PD-L1 and C) GAPDH across different experimental conditions. GAPDH was used as an experimental loading control. Independent experiments were performed at least three times and a representative blot is shown. [file 12885_2021_9009_MOESM6_ESM.docx]

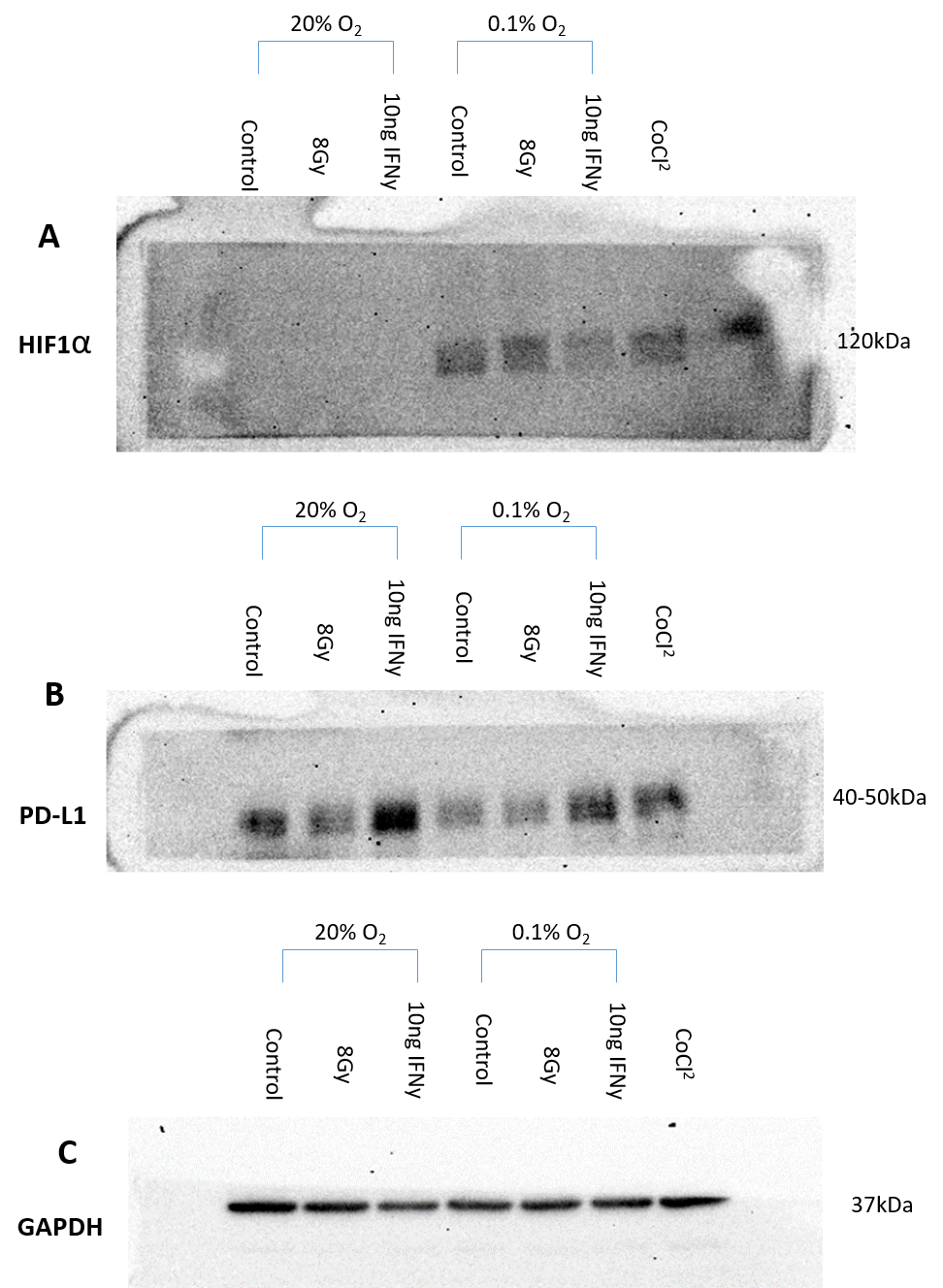


**Supplementary Figure 6. Full length original and unprocessed Western blots shown in Supplementary Figure 1.**  Western blot showing the presence/absence of **A)** HIF1a, **B)** PD-L1 and **C)** GAPDH across different experimental conditions. GAPDH was used as an experimental loading control. Independent experiments were performed at least three times and a representative blot is shown.
